# Post-release behavioral healthcare utilization and suicide risk among formerly incarcerated individuals

**DOI:** 10.1093/haschl/qxaf102

**Published:** 2025-05-17

**Authors:** Min Jang, Anne Futterer, Shari Jager-Hyman, Patrick Kessel, Jaymes Fairfax-Columbo, Suet Lim, Molly Candon

**Affiliations:** National Safety Net Advancement Center, College of Health Solutions, Arizona State University, Phoenix, AZ 85004, United States; Center for Mental Health, Department of Psychiatry, Perelman School of Medicine, University of Pennsylvania, Philadelphia, PA 19104, United States; Leonard Davis Institute of Health Economics, University of Pennsylvania, Philadelphia, PA 19104, United States; Center for Mental Health, Department of Psychiatry, Perelman School of Medicine, University of Pennsylvania, Philadelphia, PA 19104, United States; Leonard Davis Institute of Health Economics, University of Pennsylvania, Philadelphia, PA 19104, United States; Department of Psychiatry, Perelman School of Medicine, University of Pennsylvania, Philadelphia, PA 19104, United States; Philadelphia Department of Behavioral Health and Intellectual DisAbility Services, Philadelphia, PA 19107, United States; Philadelphia Department of Behavioral Health and Intellectual DisAbility Services, Philadelphia, PA 19107, United States; Philadelphia Department of Behavioral Health and Intellectual DisAbility Services, Philadelphia, PA 19107, United States; Center for Mental Health, Department of Psychiatry, Perelman School of Medicine, University of Pennsylvania, Philadelphia, PA 19104, United States; Leonard Davis Institute of Health Economics, University of Pennsylvania, Philadelphia, PA 19104, United States; Department of Health Care Management, The Wharton School, University of Pennsylvania, Philadelphia, PA 19104, United States

**Keywords:** suicide, justice-involved population, systems of care, access to care, behavioral health care, mental health, substance use disorder, community health, Medicaid

## Abstract

**Introduction:**

Individuals who experience incarceration exhibit alarmingly high rates of suicide, particularly in the immediate post-release period. This study investigates the association between post-release behavioral healthcare utilization and suicide risk among individuals released from jail.

**Methods:**

Incarceration records for 61 438 individuals released from Philadelphia County jails between 2003 and 2016 were linked with administrative datasets, including the Medical Examiner's Office, involuntary commitment petitions, Medicaid enrollment and behavioral health claims, and emergency housing episodes. Using marginal structural Cox proportional hazards models, we examined the impact of post-release service engagement on suicide risk.

**Results:**

Sixty-five percent of formerly incarcerated individuals had behavioral health diagnoses. Only 27% used outpatient mental health services post-release, with 18% of these users engaging only once, and 28% used outpatient drug and alcohol services, with 21% of these users engaging only once. Low engagement in these services was associated with higher rates of suicide attempts and deaths.

**Conclusion:**

These results underscore the critical need for comprehensive systems that ensure smooth transitions from jail-based to community care to facilitate continuous service engagement. Implementing targeted interventions and policies to improve access to and sustained engagement with behavioral healthcare services is imperative to reduce suicide risk among individuals who experience incarceration.

**Key Takeaways**

- A significant proportion (65%) of formerly incarcerated individuals had behavioral health diagnoses, but only a small percentage utilized outpatient mental health (27%) and drug and alcohol services (28%) post-release, with many engaging only once.
- Low engagement with outpatient mental health and drug and alcohol services was associated with higher rates of suicide attempts and deaths among formerly incarcerated individuals.
- The study highlights the urgent need for comprehensive systems to ensure smooth transitions from jail-based to community care and promote sustained engagement with behavioral healthcare services to reduce suicide risk in this population.

## Introduction

In the United States, over 7 million jail admissions were reported from July 2021 to June 2022, with more than 1.2 million individuals in prisons as of December 2022.^1,2^ Justice-involved populations exhibit a high prevalence of mental health and substance use disorders: according to one study, 58% of state prisoners and 63% of jail inmates meet the Diagnostic and Statistical Manual of Mental Disorders criteria for drug dependence or abuse.^3,4^ Another study found that 56% of jail inmates experienced mental health disorders in the past year, with 66% reporting substance use disorders during the same period.^5^ Alarmingly, 40%-50% of incarcerated individuals report lifetime suicide ideation, and 13%-20% have attempted suicide.^6-8^ Suicide risk among formerly incarcerated individuals is 62% higher compared with the general population.^9^

The risk for suicide is particularly acute immediately following incarceration.^10-13^ In Washington state, the risk of death from any cause during the first 2 weeks after release from prison is 12.7 times higher than that of the general population, with leading causes including drug overdose, cardiovascular disease, homicide, and suicide.^14^ In North Carolina, formerly incarcerated people face approximately 2.2 times the suicide mortality rate of the general population in the first 2 weeks post-release, and 1.9 times in the first 2 years post-release.^10^ The substantial risk for suicide in the post-incarceration period has also been documented in Australia and European countries.^11-13^

Multiple risk factors during the reentry period contribute to the high rate of suicide among formerly incarcerated individuals. Despite the prevalence of mental health and substance use disorders, many do not receive adequate treatment while incarcerated or after release. Approximately 40% of individuals with mental health conditions receive no treatment while incarcerated, and 48% of men and 59% of women with substance use disorders receive no treatment.^15^ Upon reentry, many lack health insurance, increasing their risk of requiring emergency care, hospitalization, and death.^15,16^ Financial instability often prevents returning individuals from securing safe and adequate housing, and their incarceration frequently leads to fractured social networks and strained personal relationships, further compounding post-incarceration challenges.^17^

Given the high rates of mental health and substance use disorders, lack of healthcare access, and elevated suicide risk during the reentry period, timely interventions targeting justice-involved populations are essential. However, little is known about the association between post-release behavioral healthcare use and suicide risk. Most research on service engagement for justice-involved individuals focuses on the period of incarceration rather than reentry into the community.^18^ Additionally, prior studies disproportionately examine prison populations, overlooking jails, which housed 30% of the U.S. justice-involved population in 2024.^18,19^ This distinction is critical: jails are short-term facilities operated by local governments to detain individuals awaiting trial—81% of whom have not been convicted–or serving sentences of less than a year.^19,20^ In contrast, prisons are long-term facilities for individuals convicted of serious crimes and serving extended sentences.^20-22^ For example, from July 2021 to June 2022, the average jail stay was 32 days, with a weekly turnover rate of 43%.^23^ Conversely, as of 2020, 63% of prison inmates were serving sentences of 10 years or more, and 1 in 7 was serving a life sentence.^22,24^

To address this gap, we conducted a cohort study of individuals released from Philadelphia County jails between 2003 and 2016 using large administrative datasets. Our study aims to answer 2 critical questions: (1) Is the use of behavioral healthcare associated with the risk of suicide attempts and suicide death? and (2) Is the level of service engagement associated with a reduced risk of suicide? By examining these questions, we seek to provide valuable insights that could inform more effective interventions and policies to support the mental health and well-being of justice-involved individuals during the reentry period.

## Methods

### Study sample and data sources

The study sample included individuals released from Philadelphia County jails in Pennsylvania between 2003 and 2016 who enrolled in Medicaid within 2 years after release. For those with multiple incarcerations, we used the most recent discharge as the index event. Incarceration data, obtained from the Philadelphia Department of Prisons, include entry and discharge dates but does not include information on charges, sentencing, parole, or probation. We linked this data with 2001-2018 records from the Medical Examiner's Office, involuntary commitment petitions, Medicaid enrollment and behavioral health claims, and emergency housing episodes.

Suicide attempts were identified through involuntary commitment petitions, which include a separate justification for “suicide attempt.” Suicide deaths were identified using records from the Medical Examiner's Office. Our data only include suicide attempts and deaths that occurred within Philadelphia County. Behavioral healthcare use and diagnoses were extracted from Medicaid behavioral health claims provided by Philadelphia's Community Behavioral Health. Demographic information and enrollment periods were obtained from Medicaid enrollment files. Emergency shelter stays were tracked using data from Philadelphia's Office of Homeless Services. This study was approved by the Institutional Review Boards of the City of Philadelphia and the University of Pennsylvania.

### Outcome and exposures

The primary outcome was the time from jail release to suicide attempt, with suicide death as the secondary outcome. This approach was chosen for its greater statistical power due to the higher frequency of suicide attempts. Prior suicide attempts are well-documented as one of the most robust predictors of suicide death, and our findings corroborate this.^25-28^ For example, the hazard ratio for suicide attempt history was 2.35 (95% CI = 1.10-5.04) in the service engagement model and 2.31 (95% CI = 1.09-4.90) in the service engagement level model, after adjusting for all covariates.

Behavioral healthcare service use within the first two years post-discharge was examined across 4 categories: (1) outpatient mental health, (2) outpatient drug and alcohol treatment (D&A), (3) medication management, and (4) case management, identified using procedure codes. We assessed whether engagement with these services was associated with suicide attempts and suicide deaths and how this association varied by engagement level. Exposure variables were treated as time-varying covariates. Further details are provided in the Statistical Analyses section.

### Covariates

We controlled for demographic characteristics (age, sex, race/ethnicity), incarceration-related factors (duration of incarceration whether it was the first incarceration, and year of release), and pre-discharge histories of behavioral health diagnoses, psychiatric hospitalizations, involuntary commitment petitions, and emergency shelter stays. Behavioral health diagnoses included schizophrenia, depressive disorder, bipolar disorder, substance use disorder, and other disorders, identified using an *International Statistical Classification of Disease and Related Health Problems, Tenth Revision* (ICD-10) codes.

### Statistical analyses

Cox proportional hazards regression models were used to evaluate the relationship between behavioral healthcare service utilization and time to outcomes. For individuals not experiencing involuntary commitment due to a suicide attempt (or who did not die by suicide in the suicide death model), data were censored on December 31, 2018, the end of the study period. Our model-building approach involved 3 steps: first, running unadjusted Cox models; second, adjusting for all baseline and time-varying covariates; and third, using a marginal structural model (MSM) with inverse probability of treatment weighting to adjust for time-dependent confounders.^29^ This approach accounted for within-subject correlation and provided robust variance estimators.^30,31^ Further details are provided in the Appendix.

Time-varying indicator variables were created to distinguish periods before and after first use of each behavioral healthcare service within 2 years post-discharge. Service engagement levels were categorized into low, medium, and high based on total encounters during this period: low engagement represented the bottom 25% of encounters; high engagement represented the top 25%; and medium engagement encompassed the middle 50%. Wald tests were used to examine differences in effects across these categories.

## Results

The median age of the formerly incarcerated individuals at baseline was 34 years, and 32% were female (Table 1). Non-Hispanic Black, Hispanic, non-Hispanic White, and other races made up 67.6%, 13.2%, 17.0%, and 2.2% of the population, respectively. About 56.4% had multiple incarceration episodes. Prior to the index incarceration, 4.3% had an involuntary commitment due to a suicide attempt, 18.8% had a history of depressive disorder, 41.5% had a substance use disorder, 11.5% had bipolar disorder, and 13.7% had schizophrenia. Additionally, 47.9% had other behavioral health disorders, 19.2% had a history of psychiatric hospitalization, and 13.6% used emergency shelter services. The median follow-up time was 63 months (IQR: 37, 105) for suicide attempts and 64 months (IQR: 39, 107) for suicide deaths, both from release until the event or censoring. After release from jail, 3.0% (*n* = 1074) had an involuntary commitment indicating a suicide attempt, and 0.2% (*n* = 77) died by suicide.

**Table 1.** Baseline characteristics, 2001-2016.

Variable	%/N
Demographic characteristics	
Female	32.0%/11 560
Race/ethnicity	
Black	67.6%/24 415
Hispanic	13.2%/4767
White	17.0%/6152
Others	2.2%/800
Age	
10-17	1.5%/529
18-24	20.2%/7286
25-39	42.6%/15 399
40+	35.8%/12 920
Index incarceration characteristics	
First jail incarceration	43.6%/15 758
Number of days in jail	
≤ 3 days	27.4%/9890
≤ 20 days	23.1%/8363
≤ 100 days	23.6%/8522
100 + days	25.9%/9359
Other characteristics: prior to the index incarceration	
Behavioral health	
Any behavioral health disorder	65.2%/23 574
Schizophrenia	13.7%/4938
Depressive disorder	18.8%/6802
Bipolar disorder	11.5%/4156
Substance use disorder	41.5%/14 999
Other behavioral health disorders	47.9%/17 297
Emergency shelter use	13.6%/4899
Involuntary commitment claim	
Suicide attempt	4.3%/1549
Others	9.2%/3331
Psychiatric hospitalization	19.2%/6950
N	36 134

**Source**: Authors’ analysis of Philadelphia Medicaid-funded behavioral health claims data, records of emergency housing episodes, involuntary commitment petitions, and incarceration episodes for individuals released from Philadelphia County jails between 2003 and 2016 who enrolled in Medicaid within two years of release.

For individuals with multiple incarcerations, the most recent incarceration was designated as the index incarceration. Individuals were identified as having a diagnosis if they had any claims before release with an International Statistical Classification of Disease and Related Health Problems, Tenth Revision (ICD-10) code for each diagnosis.

Table 2 summarizes the use of behavioral healthcare services during the 2 years after release. Outpatient mental health services were used by 27.4% of individuals, with mean encounters of 1.4, 8.0, and 43.8 for low, medium, and high-intensity users, respectively. Outpatient D&A services were used by 27.8%, with mean encounters of 1.3, 12.1, and 64.7 for low, medium, and high-intensity users. Case management services were used by 6.5%, with mean encounters of 3.2, 21.3, and 113.6 for low, medium, and high-intensity users. Medication management services were used by 19.9%, with mean encounters of 1.4, 5.4, and 17.1 for low, medium, and high-intensity users.

**Table 2.** Post-release behavioral health service encounters by engagement level, 2003-2018.

Behavioral health service	Any use %/N	Mean number of encounters/SD		
	full sample	Users with low engagement	Users with medium engagement	Users with high engagement
Outpatient mental health	27.4%/9900	1.4 (0.5)	8.0 (4.4)	43.8 (26.2)
Outpatient D&A	27.8%/10 030	1.3 (0.5)	12.1 (7.6)	64.7 (40.2)
Case management	6.5%/2349	3.2 (1.8)	21.3 (11.4)	113.6 (65.8)
Medication management	19.9%/7172	1.4 (0.5)	5.4 (2.0)	17.1 (9.2)

**Source**: Authors’ analysis of Philadelphia Medicaid-funded behavioral health claims data and incarceration episodes for individuals released from Philadelphia County jails between 2003 and 2016 who enrolled in Medicaid within two years of release.

Service engagement levels were categorized as low, medium, and high based on total encounters during the first two years after release. Low engagement represented the bottom 25% of users by encounter frequency, high engagement the top 25%, and medium engagement the middle 50%.

### Association between service engagement and suicide attempt/death

#### Suicide attempt

Table 3 presents hazard ratios (HRs) for involuntary commitments indicating a suicide attempt for each service exposure, estimated by 3 different models. In unadjusted analyses, the use of outpatient mental health, medication management, and case management services after discharge was associated with a higher risk of suicide attempt. Compared with non-users, individuals who used outpatient mental health services had a 2.15-fold higher risk of suicide attempt (95% CI = 1.83-2.52). Those who used medication management services had a 1.20-fold higher risk (95% CI = 1.01-1.43), while case management service users had a 2.27-fold higher risk (95% CI = 1.86-2.77).

**Table 3.** Association between post-release behavioral healthcare service use and hazard of suicide attempt, 2001-2018.

	HR (95% CI)			
Model	Outpatient mental health	D&A	Medication management	Case management
Unadjusted model	2.15 (1.83 to 2.52)	1.14 (0.98 to 1.32)	1.20 (1.01 to 1.43)	2.27 (1.86 to 2.77)
Adjusted model	1.32 (1.13 to 1.54)	0.98 (0.84 to 1.14)	0.87 (0.73 to 1.03)	1.23 (1.01 to 1.50)
Structural model	1.06 (0.91 to 1.25)	0.87 (0.74 to 1.01)	0.76 (0.65 to 0.90)	1.19 (0.97 to 1.47)

Source: Authors’ analysis of Philadelphia Medicaid-funded behavioral health claims data, records of emergency housing episodes, involuntary commitment petitions, and incarceration episodes for individuals released from Philadelphia County jails between 2003 and 2016 who enrolled in Medicaid within two years of release.

Hazard ratios (HRs) for involuntary commitments indicating suicide attempts are presented for each service exposure. HRs were estimated using 3 models: (1) unadjusted Cox models, (2) Cox models adjusted for baseline and time-varying covariates, and (3) marginal structural Cox models with inverse probability of treatment weighting to adjust for time-dependent confounders. The number of person-month observations is 2 638 343 (36 134 formerly incarcerated individuals).

Controlling for covariates attenuated these associations, resulting in lower HRs and reduced statistical significance: 1.32 (95% CI = 1.13-1.54) for outpatient mental health services, 0.87 (95% CI = 0.73-1.03) for medication management services, and 1.23 (95% CI = 1.01-1.50) for case management services. Adjustment for time-dependent confounders further attenuated the associations, resulting in a significantly lower risk of suicide attempt for medication management service users compared with non-users. The HRs were 1.06 (95% CI = 0.91-1.25) for outpatient mental health services, 0.76 (95% CI = 0.65-0.90) for medication management services, and 1.19 (95% CI = 0.97-1.47) for case management services. No significant association was found between D&A services and suicide attempts.

#### Suicide death

Appendix Table S1 presents the HRs for suicide death for each service exposure. In unadjusted analyses, outpatient mental health service use was associated with a 1.96-fold higher risk of suicide (95% CI = 1.08-3.55). Controlling for covariates resulted in a lower HR and reduced statistical significance (HR = 1.83; 95% CI = 0.98-3.43). Adjusting for time-dependent confounders further attenuated the association, with the HR decreasing to 1.61 (95% CI = 0.88-2.94). No significant associations were found between other services and suicide death across all models.

### Differential association by service engagement level

**Figure 1.**
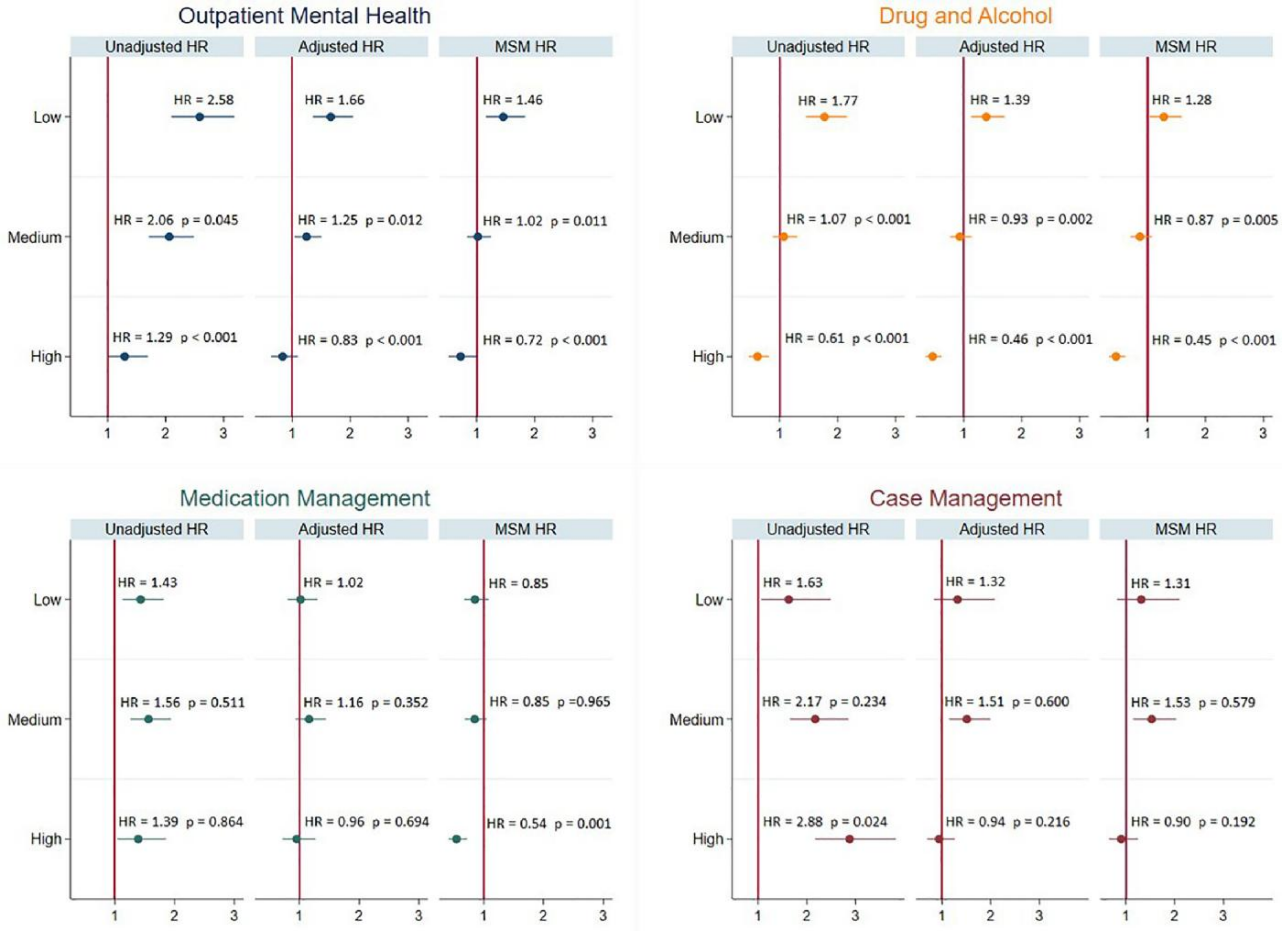
Association between post-release behavioral healthcare service use and hazard of suicide attempt by engagement level, 2001-2018. Source: Authors’ analysis of Philadelphia Medicaid-funded behavioral health claims data, records of emergency housing episodes, involuntary commitment petitions, and incarceration episodes for individuals released from Philadelphia County jails between 2003 and 2016 who enrolled in Medicaid within 2 years of release. Notes: Hazard ratios (HRs) for involuntary commitments indicating suicide attempts are presented for each service exposure. HRs were estimated using 3 models: (1) unadjusted Cox models, (2) Cox models adjusted for baseline and time-varying covariates, and (3) marginal structural Cox models with inverse probability of treatment weighting to adjust for time-dependent confounders. The *P* values shown are from Wald tests of the null hypothesis that the hazard ratio for medium/high engagement is equal to that for low engagement. The number of person-month observations is 2 638 343 (36 134 formerly incarcerated individuals).

We examined whether these associations varied by level of service engagement. Figure 1 and Appendix Figure S1 present results for suicide attempts and deaths, respectively, by service engagement level.

#### Suicide attempt

##### Outpatient mental health service

In unadjusted analyses, both high (*P* < 0.001) and medium (*P* = 0.045) service engagements were associated with lower rates of suicide attempts compared with low engagement. Any level of service use was associated with higher suicide attempt rates compared with non-engagement (low: HR = 2.58, 95% CI = 2.10-3.18; medium: HR = 2.06, 95% CI = 1.71-2.49; high: HR = 1.29, 95% CI = 0.99-1.69). The differences by engagement level remained significant after adjusting for covariates (*P* = 0.012 for medium vs low, *P* < 0.001 for high vs low), with HRs of 1.66 (95% CI = 1.35-2.05) for low engagement, 1.25 (95% CI = 1.03-1.50) for medium engagement, and 0.83 (95% CI = 0.64-1.10) for high engagement. These differences also persisted after adjusting for time-dependent confounders (*P* = 0.011 for medium vs low, *P* < 0.001 for high vs low), with HRs of 1.46 (95% CI = 1.16-1.83) for low engagement, 1.02 (95% CI = 0.83-1.25) for medium engagement, and 0.72 (95% CI = 0.51-1.02) for high engagement. Notably, HRs for high engagement fell below 1 in these adjusted models.

##### D&A service

In unadjusted analyses, both high and medium engagement were associated with lower rates of suicide attempts compared with low engagement (*P* < 0.001 for both medium vs low and high vs low), with HRs of 1.77 for low engagement (95% CI = 1.45-2.16), 1.07 for medium engagement (95% CI = 0.88-1.30), and 0.61 for high engagement (95% CI = 0.46-0.82). The differences by engagement level remained significant after adjusting for covariates (*P* = 0.002 for medium vs low, *P* < 0.001 for high vs low) with HRs of 1.39 (95% CI = 1.13-1.70) for low engagement, 0.93 (95% CI = 0.76-1.14) for medium engagement, and 0.46 (95% CI = 0.35-0.62) for high engagement. These differences also persisted after adjusting for time-dependent confounders (*P* = 0.005 for medium vs low, *P* < 0.001 for high vs low) with HRs 1.28 (95% CI = 1.03-1.59 for low engagement, 0.87 (95% CI = 0.70-1.07) for medium engagement, and 0.45 (95% CI = 0.33-0.62) for high engagement. Notably, high engagement was significantly associated with a lower suicide attempt rate compared with non-engagement across all models.

##### Medication management service

No significant difference in suicide attempt risk by engagement level was found in the first 2 models. After adjusting for time-dependent confounders, high engagement was associated with a lower rate of suicide attempts compared with low engagement (*P* = 0.001) and non-engagement, with HRs of 0.85 for low engagement (95% CI = 0.67-1.08) and 0.54 for high engagement (95% CI = 0.41-0.72).

##### Case management service

In unadjusted analyses, high engagement was associated with a higher rate of suicide attempts compared with low engagement (*P* = 0.024). Any level of service use was associated with higher suicide attempt rates compared with non-engagement (low: HR = 1.63, 95% CI = 1.06-2.49; medium: HR = 2.17, 95% CI = 1.65-2.85; high: HR = 2.88, 95% CI = 2.17-3.82). This association reversed in adjusted models with high engagement showing lower HRs (though insignificant) than low engagement.

#### Suicide death

In unadjusted analyses, high engagement with outpatient mental health and D&A services was associated with lower rates of suicide deaths compared with low engagement (*P* = 0.034 for mental health; *P* = 0.012 for D&A), with HRs of 3.02 (95% CI = 1.54-5.92) and 0.92 (95% CI = 0.30-2.76) for low and high outpatient mental health engagement, respectively, and HRs of 2.49 (95% CI = 1.25-4.97) and 0.61 (95% CI = 0.21-1.75) for low and high D&A engagement, respectively. For D&A, the difference between high and low engagement remained significant after adjusting for covariates (*P* = 0.008; low: HR = 2.11, 95% CI = 1.04-4.28; high: HR = 0.45, 95% CI = 0.14-1.42) and for time-dependent confounders (*P* < 0.001; low: HR = 2.06, 95% CI = 1.08-3.93; high: HR = 0.26, 95% CI = 0.08-0.79). For outpatient mental health, the difference remained significant after adjusting for covariates (*P* = 0.031; low: HR = 2.87, 95% CI = 1.46-5.67; high: HR = 0.86, 95% CI = 0.29-2.57) and became insignificant after adjusting for time-dependent confounders (*P* = 0.376; low: HR = 2.31, 95% CI = 1.16-4.60; high: HR = 1.39, 95% CI = 0.48-4.03). Across all models, low engagement with outpatient mental health and D&A services was associated with higher suicide death rates than non-engagement. No significant difference in suicide death risk by engagement level was found for medication management and case management services.

## Discussion

In this retrospective study of individuals released from jail in Philadelphia County, we found that high engagement with outpatient mental health or D&A services was associated with lower rates of suicide attempts compared with low engagement. This pattern was also observed for suicide deaths, with a significant difference between low and high engagement. While our results suggest more frequent service engagement after release from jail is linked to a lower rate of suicidal behaviors, the formerly incarcerated population may suffer from underutilization of behavioral healthcare services. In our sample, 65.2% of the formerly incarcerated individuals had pre-existing behavioral health issues, but only 27.4% ever used outpatient mental health services post-release, with 17.8% of these users accessing the service only once. Similarly, 41.5% had substance use disorders, but only 27.8% ever used D&A services post-release, with 20.9% of these users accessing the service only once.

Past studies suggest that rapid initiation of treatment following discharge is critical.^10-13,32-34^ Our study further emphasizes the importance of treatment frequency, underscoring the urgent need for comprehensive systems to support the mental health of formerly incarcerated individuals. Unfortunately, many barriers may make engaging in treatment upon reentry challenging.^35^ To this end, the criminal justice and behavioral health systems should work to facilitate efficient transition from jail-based to community care and continuous service engagement.^36^ This includes navigating insurance gaps by facilitating enrollment in Medicaid benefits for returning citizens who are uninsured, or facilitating resumption for individuals whose benefits had been suspended.^35^ Additionally, targeted interventions should address the specific needs of this population, improve access to comprehensive behavioral healthcare services, and implement policies that support sustained engagement.

This study has several limitations. First, our claims data include only services paid for by Community Behavioral Health (CBH), Philadelphia's sole behavioral health Medicaid managed care organization. As a result, services provided outside CBH's scope—such as care received outside Philadelphia or through other insurance types—are not captured, which may bias our estimates, including those of service use rates. Second, we did not have data on whether individuals were released to the community or transferred to another facility, including prisons, after their jail stay. This limitation prevented us from assessing how different post-release settings affect access to care, service engagement, and suicide risk. Third, relying on involuntary commitment petition records to identify suicide attempts may result in underreporting. Fourth, despite extensive covariate adjustment, residual confounding likely persists, especially since lifetime behavioral health indicators may not reflect acute conditions or symptom severity at release. Fifth, we included only suicide attempts and deaths that occurred within Philadelphia County, so individuals who attempted or died by suicide outside the county were not captured. This may lead to underestimation of suicide outcomes; for example, if someone moved out of the county after release and died by suicide elsewhere, their death would not be included.

Lastly, our focus on Medicaid-eligible individuals released from Philadelphia jails limits generalizability. While a large proportion of incarcerated individuals are eligible for Medicaid—80% to 90% in Medicaid expansion states—we included only those enrolled within 2 years after release.^37-39^ This criterion may not represent the entire jail-released population, especially given the challenges of enrolling during reentry. In our data, 46% (30 661 of 66 795) of Medicaid-eligible individuals (those enrolled at any point post-release) did not enroll within 2 years, indicating our sample may exclude a significant portion of those eligible. If those who did not enroll had a higher suicide risk than those who enrolled, our estimates for the effect of service use on reducing suicide risk might be underestimated. Future research should extend to diverse populations across various geographic areas and healthcare systems and incorporate more comprehensive measures of mental health at release for accurate risk assessment.

## Supplementary Material

qxaf102_Supplementary_Data

## References

[1] Zeng Z . Jails Report Series: Preliminary Data Release. Bureau of Justice Statistics, US Department of Justice; September 2023. Accessed June 26, 2024. https://bjs.ojp.gov/library/publications/jails-report-series-preliminary-data-release-2022#:∼:text=Jails%20reported%207.3%20million%20admissions,14%25%20of%20the%20inmate%20population

[2] Carson EA, Kluckow R. Prisoners in 2022—Statistical Tables. Bureau of Justice Statistics, US Department of Justice; November 2023. Accessed June 24, 2024. https://bjs.ojp.gov/library/publications/prisoners-2022-statistical-tables

[3] American Psychiatric Association . Diagnostic and Statistical Manual of Mental Disorders. 4th ed. American Psychiatric Publishing, Inc; 1994.

[4] Bronson J, Stroop J, Zimmer S, Berzofsky M. Drug use, Dependence, and Abuse among State Prisoners and Jail Inmates, 2007-2009. Bureau of Justice Statistics, US Department of Justice; June 2017. Accessed June 26, 2024. https://bjs.ojp.gov/content/pub/pdf/dudaspji0709.pdf

[5] James D, Glaze LE. Mental health problems of prison and jail inmates. Bur Justice Stat Spec Rep. 2006. Accessed May 21, 2025, https://bjs.ojp.gov/content/pub/pdf/mhppji.pdf

[6] Hayes LM, Rowan JR. National Study of Jail Suicides: Seven Years Later. National Center for Institutions and Alternatives; 1988.

[7] Charles DR, Abram KM, McClelland GM, Teplin LA. Suicidal ideation and behavior among women in jail. J Contemp Crim Justice. 2003;19(1):65–81. 10.1177/1043986202239742

[8] Sarchiapone M, Jovanovic N, Roy A, et al Relations of psychological characteristics to suicide behavior: results from a large sample of male prisoners. Personal. Individ. Differ. 2009;47(4):250–255. 10.1016/j.paid.2009.03.008

[9] Morgan ER, Rivara FP, Ta M, Grossman DC, Jones K, Rowhani-Rahbar A. Incarceration and subsequent risk of suicide: a statewide cohort study. Suicide Life Threat Behav. 2022;52(3):467–477. 10.1111/sltb.12834 35092087

[10] Fitch KV, Pence BW, Rosen DL, et al Suicide mortality among formerly incarcerated people compared with the general population in North Carolina, 2000–2020. Am JEpidemiol. 2024;193(3):489–499. 10.1093/aje/kwad214 37939151 PMC11484614

[11] Bukten A, Stavseth MR. Suicide in prison and after release: a 17-year national cohort study. Eur J Epidemiol. 2021;36(10):1075–1083. 10.1007/s10654-021-00782-0 34427828 PMC8542551

[12] Pratt D, Piper M, Appleby L, Webb R, Shaw J. Suicide in recently released prisoners: a population-based cohort study. Lancet. 2006;368(9530):119–123. 10.1016/S0140-6736(06)69002-8 16829295

[13] Kariminia A, Law MG, Butler TG, et al Suicide risk among recently released prisoners in New South Wales, Australia. Med J Aust. 2007;187(7):387–390. 10.5694/j.1326-5377.2007.tb01307.x 17908000

[14] Binswanger IA, Stern MF, Deyo RA, et al Release from prison—a high risk of death for former inmates. N Engl J Med. 2007;356(2):157–165. 10.1056/NEJMsa064115 17215533 PMC2836121

[15] Mallik-Kane K, Visher CA. Health and Prisoner Reentry: how Physical, Mental, and Substance Abuse Conditions Shape the Process of Integration. Urban Institute; 2008. Accessed July 3, 2024. https://www.urban.org/sites/default/files/publication/31491/411617-Health-and-Prisoner-Reentry.PDF

[16] Rich JD, Chandler R, Williams BA, et al How health care reform can transform the health of criminal justice-involved individuals. Health Aff(Millwood). 2014;33(3):462–467. 10.1377/hlthaff.2013.1133 24590946 PMC4034754

[17] Herman-Stahl M, Kan ML, McKay T. Incarceration and the Family: A Review of Research and Promising Approaches for Service Fathers and Families. US Department of Health and Human Services; 2008. Accessed July 3, 2024. https://aspe.hhs.gov/sites/default/files/migrated_legacy_files//42886/report.pdf

[18] Carter A, Butler A, Willoughby M, et al Interventions to reduce suicidal thoughts and behaviours among people in contact with the criminal justice system: a global systematic review. EClinicalMedicine. 2022;44:101266. 10.1016/j.eclinm.2021.101266 35072018 PMC8763634

[19] Sawyer W, Wagner P. Mass Incarceration: The Whole Pie 2024. Prison Policy Initiative; March 14, 2024. Accessed April 7, 2025. https://www.prisonpolicy.org/reports/pie2024.html

[20] Schlanger M, Bedi S, Shapiro D. Incarceration and the Law: Cases and Materials (Table of Contents and Ch. 1 Excerpt). West Academic Publishing; 2020. https://ssrn.com/abstract=3802905

[21] Nellis A . No End in Sight: America's Enduring Reliance on Life Imprisonment. The Sentencing Project; 2021. https://perma.cc/PB2R-8F9U

[22] Council on Criminal Justice . Long sentences by the numbers. 2022. Accessed May 21, 2025. https://counciloncj.foleon.com/tfls/long-sentences-by-thenumbers/Long-Sentences-Behind-Bars

[23] Zeng Z . Jail Inmates in 2022—Statistical Tables (NCJ 307086). Bureau of Justice Statistics, U.S. Department of Justice; December 2023.

[24] Nellis A . No End in Sight: America's Enduring Reliance on Life Imprisonment. Sentencing Project; 2021. https://www.sentencingproject.org/app/uploads/2022/08/No-End-in-Sight-Americas-Enduring-Reliance-on-Life-Imprisonment.pdf

[25] Bostwick JM, Pabbati C, Geske JR, McKean AJ. Suicide attempt as a risk factor for completed suicide: even more lethal than we knew. Am J Psychiatry. 2016;173(11):1094–1100. 10.1176/appi.ajp.2016.15070854 27523496 PMC5510596

[26] Suominen K, Isometsä E, Suokas J, Haukka J, Achte K, Lönnqvist J. Completed suicide after a suicide attempt: a 37-year follow-up study. Am J Psychiatry. 2004;161(3):562–563. 10.1176/appi.ajp.161.3.562 14992984

[27] Park S, Lee Y, Youn T, et al Association between level of suicide risk, characteristics of suicide attempts, and mental disorders among suicide attempters. BMC Public Health. 2018;18(1):477. 10.1186/s12889-018-5387-8 29642887 PMC5896087

[28] Lin IL, Tseng JY, Tung HT, Hu YH, You ZH. Predicting the risk of future multiple suicide attempt among first-time suicide attempters: implications for suicide prevention policy. Healthcare(Basel). 2022;10(4):66723. 10.3390/healthcare10040667 PMC9032869 35455845

[29] Robins JM, Hernan MA, Brumback B. Marginal structural models and causal inference in epidemiology. Epidemiology. 2000;11(5):550–560. 10.1097/00001648-200009000-00011 10955408

[30] Hern´an MA, Brumback BA, Robins JM. Estimating the causal effect of zidovudine on CD4 count with a marginal structural model for repeated measures. Stat Med. 2002;21(12):1689–1709. 10.1002/sim.1144 12111906

[31] Xiao Y, Abrahamowicz M, Moodie EE. Accuracy of conventional and marginal structural cox model estimators: a simulation study. Int J Biostat. 2010;6(2):13. 10.2202/1557-4679.1208 21969997

[32] Van Dorn RA, Desmarais SL, Petrila J, Haynes D, Singh JP. Effects of outpatient treatment on risk of arrest of adults with serious mental illness and associated costs. Psychiatr Serv. 2013;64(9):856–862. 10.1176/appi.ps.201200406 23677480

[33] Thomas EG, Spittal MJ, Taxman FS, Puljević C, Heffernan EB, Kinner SA. Association between contact with mental health and substance use services and reincarceration after release from prison. PLoS One. 2022;17(9):e0272870. 10.1371/journal.pone.0272870 36070251 PMC9451082

[34] Substance Abuse and Mental Health Services Administration . Guidelines for Successful Transition of People with Mental or Substance use Disorders from Jail and Prison: Implementation Guide. SAMHSA; 2017. Accessed October 1, 2024. https://library.samhsa.gov/sites/default/files/sma16-4998.pdf

[35] Fahmy C, Wallace D. Incarceration, reentry, and health. In: Huebner BM, Frost NA, eds. Handbook on the Consequences of Sentencing and Punishment Decisions. Routledge; 2018:105–121.

[36] American Psychiatric Association . Psychiatric Services in Jails and Prisons. 3rd ed., American Psychiatric Publishing, Inc; 2016.

[37] Howard J, Solan M, Neptune J, Mellgren L, Dubenitz J, Avery K. The importance of Medicaid coverage for criminal justice involved individuals reentering their communities. April 2016. Accessed April 15, 2025. https://aspe.hhs.gov/system/files/pdf/201476/MedicaidJustice.pdf

[38] Medicaid and CHIP Payment and Access Commission . Access to medicaid coverage and care for adults leaving incarceration (chapter 3), eds. Report to Congress on Medicaid and CHIP. DC: MACPAC; June 2023:66–89. https://www.macpac.gov/wp-content/uploads/2023/06/Chapter-3-Access-to-Medicaid-Coverage-and-Care-for-Adults-Leaving-Incarceration.pdf

[39] Ryan J, Pagel L, Smali K, Artiga S, Rudowitz R, Gates A. Connecting the Justice-Involved Population to Medicaid Coverage and Care: Findings from Three States. Kaiser Family Foundation; June 2016. https://www.kff.org/report-section/connecting-the-justice-involved-population-to-medicaid-coverage-and-care-issue-brief/

