# Supplementary material for: Post-release behavioral healthcare utilization and suicide risk among formerly incarcerated individuals
Source: Health Aff Sch. 2025 May 17;3(6):qxaf102. doi: 10.1093/haschl/qxaf102 (PMC12128921; doi:10.1093/haschl/qxaf102)
Supplement: qxaf102_Supplementary_Data [file qxaf102_supplementary_data.zip › Appendix.docx]

**Appendix**

**Appendix Table 1.** Association between Post-release Behavioral Healthcare Service Use and Hazard of Suicide Death, 2001-2018

|  | **HR (95% CI)** | | | |
| --- | --- | --- | --- | --- |
| **Model** | **Outpatient mental health** | **D&A** | **Medication management** | **Case management** |
| Unadjusted model | 1.96 (1.08 to 3.55) | 1.59 (0.92 to 2.75) | 1.14 (0.59 to 2.20) | 0.64 (0.23 to 1.81) |
| Adjusted model | 1.83 (0.98 to 3.43) | 1.29 (0.70 to 2.38) | 0.77 (0.39 to 1.52) | 0.58 (0.20 to 1.69) |
| Structural model | 1.61 (0.88 to 2.94) | 1.10 (0.63 to 1.93) | 0.99 (0.54 to 1.80) | 0.58 (0.17 to 1.93) |

**Source**: Authors’ analysis of Philadelphia Medicaid-funded behavioral health claims data Medical Examiner’s death records, records of emergency housing episodes, involuntary commitment petitions, and incarceration episodes for individuals released from Philadelphia County jails between 2003 and 2016 who enrolled in Medicaid within two years of release. **Notes**: Hazard ratios (HRs) for suicide deaths are presented for each service exposure. HRs were estimated using three models: 1) unadjusted Cox models, 2) Cox models adjusted for baseline and time-varying covariates, and 3) marginal structural Cox models with inverse probability of treatment weighting to adjust for time-dependent confounders.

**Appendix Figure 1.** Association between Post-release Behavioral Healthcare Service Use and Hazard of Suicide Death by Engagement Level, 2001-2018


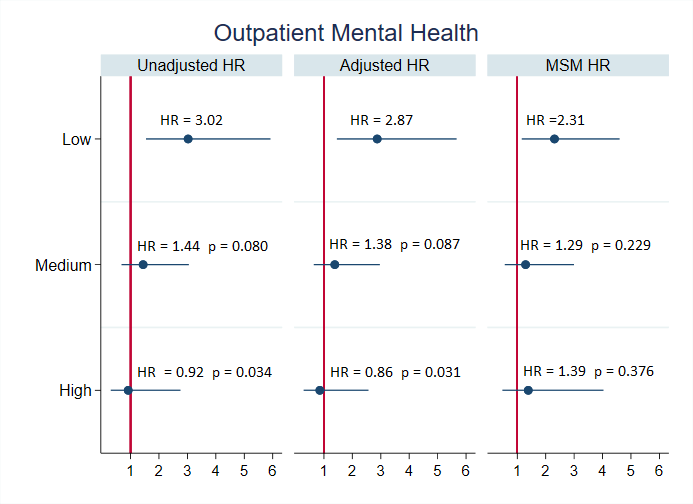

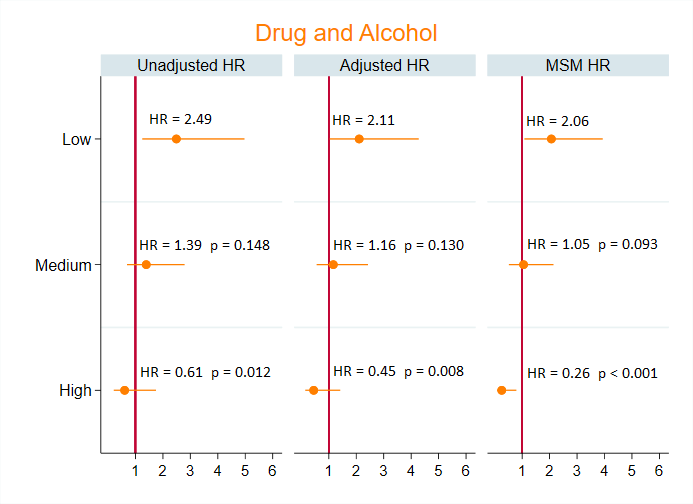

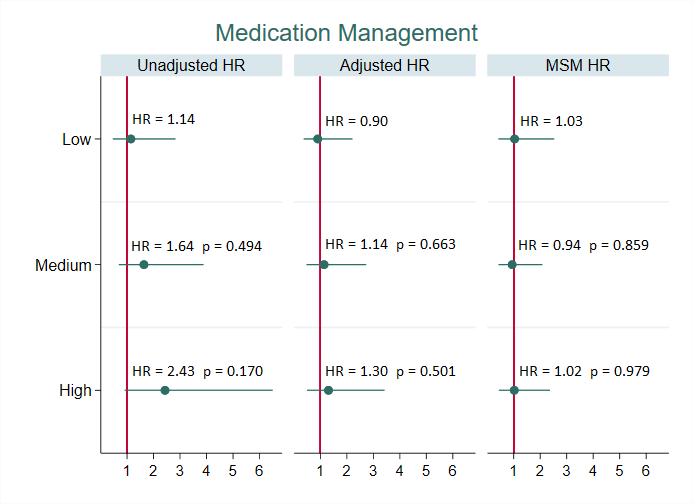

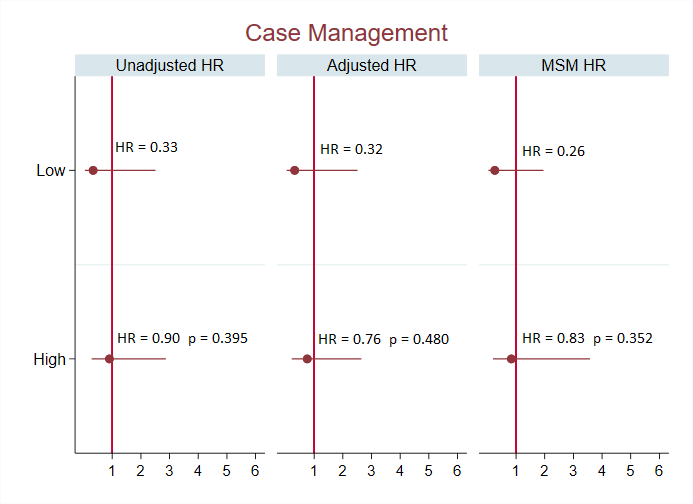


**Source**: Authors’ analysis of Philadelphia Medicaid-funded behavioral health claims data, Medical Examiner’s death records, records of emergency housing episodes, involuntary commitment petitions, and incarceration episodes for individuals released from Philadelphia County jails between 2003 and 2016 who enrolled in Medicaid within two years of release. **Notes**: Hazard ratios (HRs) for suicide deaths are presented for each service exposure. HRs were estimated using three models: 1) unadjusted Cox models, 2) Cox models adjusted for baseline and time-varying covariates, and 3) marginal structural Cox models with inverse probability of treatment weighting to adjust for time-dependent confounders. The p values shown are from Wald tests of the null hypothesis that the HR for medium/high engagement is equal to that for low engagement. For case management, the levels of engagement were categorized into low and high, excluding medium due to the limited number of suicide death.

***Marginal structural model with inverse probability of treatment weighting***

To address potential time-varying confounders, we employed a marginal structural model (MSM) with inverse probability of treatment weighting. This approach can effectively manage confounders that simultaneously influence future treatment and mediate the effects of past treatment on the outcome.^1^ In our context, behavioral healthcare need serves as such a confounder. It potentially increases the likelihood of both future behavioral healthcare use and suicidal behaviors, while being influenced by past healthcare receipt. Additionally, behavioral health treatment may reduce future care needs, subsequently lowering suicidal risk. This creates a complex relationship where the confounder acts as both a predictor and a mediator.

To address this issue, we created a weighted pseudopopulation using inverse probability of treatment weighting. In this pseudopopulation, the exposure becomes unconfounded by time-varying covariates, allowing for more accurate estimation of causal relationships.^1^ We obtained inverse probability of treatment weights (IPTW) using the approach described by Hernán et al. (2000).^2^ All estimations were performed using Stata version 17.0, following the methodology outlined by Fewell et al. (2004).^3^ Our process involved implementing logistic regression models at the person-month level for each service type, using service use as a binary outcome. We estimated the weight denominator using baseline covariates, time-varying covariates, and time variables, while calculating the numerator using only baseline covariates and time variables. We incorporated time variables, specifically the number of months since jail discharge and natural cubic splines, to address varying probabilities of using services over time.

To account for potential censoring two years after jail discharges, which could end the study period before service initiation or suicidal behaviors, we estimated inverse-probability-of-censoring weights. This process mirrored the IPTW calculation but used an indicator variable for service use censoring in the current month as the binary outcome. Finally, we weighted each observation by the product of its IPTW and inverse-probability-of-censoring weight.

**References**

1. Robins, J.M., M.A. Hern´an, and B. Brumback. 2000. Marginal structural models and causal inference in epidemiology. Epidemiology 11(5): 550–560.
2. Hern´an, M.A., B. Brumback, and J.M. Robins. 2000. Marginal structural models to estimate the causal effect of zidovudine on the survival of HIV-positive men. Epidemiology 11(5): 561–570.
3. Fewell, Z., M.A. Hern´an, F. Wolfe et al. 2004. Controlling for time-dependent confounding using marginal structural models. Stata J 4:402–420.
